# An Open-Source Privacy-Preserving Large-Scale Mobile Framework for Cardiovascular Health Monitoring and Intervention Planning With an Urban African American Population of Young Adults: User-Centered Design Approach

**DOI:** 10.2196/25444

**Published:** 2022-01-11

**Authors:** Gari Clifford, Tony Nguyen, Corey Shaw, Brittney Newton, Sherilyn Francis, Mohsen Salari, Chad Evans, Camara Jones, Tabia Henry Akintobi, Herman Taylor Jr

**Affiliations:** 1 Department of Biomedical Informatics Emory University Atlanta, GA United States; 2 Department of Biomedical Engineering Georgia Institute of Technology Atlanta, GA United States; 3 Nucleus Health Communications Atlanta, GA United States; 4 School of Interactive Computing Georgia Institute of Technology Atlanta, GA United States; 5 Cardiovascular Research Institute Morehouse School of Medicine Atlanta, GA United States; 6 Prevention Research Center & Community Engagement Morehouse School of Medicine Atlanta, GA United States

**Keywords:** agile design, cardiovascular disease, community-based participatory research, exposome, user-centered design, minority health, African American, mobile phone

## Abstract

**Background:**

Cardiovascular diseases (CVDs) are the leading cause of death worldwide and are increasingly affecting younger populations, particularly African Americans in the southern United States. Access to preventive and therapeutic services, biological factors, and social determinants of health (ie, structural racism, resource limitation, residential segregation, and discriminatory practices) all combine to exacerbate health inequities and their resultant disparities in morbidity and mortality. These factors manifest early in life and have been shown to impact health trajectories into adulthood. Early detection of and intervention in emerging risk offers the best hope for preventing race-based differences in adult diseases. However, young-adult populations are notoriously difficult to recruit and retain, often because of a lack of knowledge of personal risk and a low level of concern for long-term health outcomes.

**Objective:**

This study aims to develop a system design for the MOYO mobile platform. Further, we seek to addresses the challenge of primordial prevention in a young, at-risk population (ie, Southern-urban African Americans).

**Methods:**

Urban African Americans, aged 18 to 29 years (n=505), participated in a series of co-design sessions to develop MOYO prototypes (ie, HealthTech Events). During the sessions, participants were orientated to the issues of CVD risk health disparities and then tasked with wireframing prototype screens depicting app features that they considered desirable. All 297 prototype screens were subsequently analyzed using NVivo 12 (QSR International), a qualitative analysis software. Using the grounded theory approach, an open-coding method was applied to a subset of data, approximately 20% (5/25), or 5 complete prototypes, to identify the dominant themes among the prototypes. To ensure intercoder reliability, 2 research team members analyzed the same subset of data.

**Results:**

Overall, 9 dominant design requirements emerged from the qualitative analysis: customization, incentive motivation, social engagement, awareness, education, or recommendations, behavior tracking, location services, access to health professionals, data user agreements, and health assessment. This led to the development of a cross-platform app through an agile design process to collect standardized health surveys, narratives, geolocated pollution, weather, food desert exposure data, physical activity, social networks, and physiology through point-of-care devices. A Health Insurance Portability and Accountability Act–compliant cloud infrastructure was developed to collect, process, and review data, as well as generate alerts to allow automated signal processing and machine learning on the data to produce critical alerts. Integration with wearables and electronic health records via fast health care interoperability resources was implemented.

**Conclusions:**

The MOYO mobile platform provides a comprehensive health and exposure monitoring system that allows for a broad range of compliance, from passive background monitoring to active self-reporting. These study findings support the notion that African Americans should be meaningfully involved in designing technologies that are developed to improve CVD outcomes in African American communities.

## Introduction

### Background

Longitudinal cohort studies require significant resources to track patients over long periods, particularly for chronic diseases. Portable digital identities, such as phone numbers and emails, however, can often persist for many years, with almost half of adults living in urban areas in the United States possessing a cellphone number from elsewhere [1]. This provides an exciting possibility for recruiting and retaining electronic cohorts. The rise of mobile phone use and the complexity of sensors they possess further stretch the options of the type of data we might gather concerning a person’s health, particularly beyond standard metrics such as symptoms, weight, and blood pressure. Moreover, society is becoming increasingly aware that the environment or *exposure* of an individual will significantly influence their health trajectory [2]. However, measuring environmental exposures and behaviors over long periods is cumbersome and costly. Smartphone apps, with geolocation and intuitive interfaces for rapid ecological momentary assessment, provide a scalable and straightforward way to capture exposure data through application programming interfaces (APIs) to a more extensive infrastructure, such as weather and pollution monitoring stations, as well as wirelessly connected wearables and point-of-care devices.

Although consumer wearables have generated much excitement over the last decade, they have from 2 fundamental problems. First, they do not provide access to the raw data. Conversely, wearables provide access to some undisclosed, and often evolving, proprietary metrics or nonstandard measures such as *steps*. Mobile phones, particularly Android-based ones, do in fact provide such access and can allow a user to extract deep, actionable insights from their data [3,4]. Second, the lack of *stickiness* among wearables is a significant problem, with over half of the wearable consumer base ceasing to wear a fitness tracker after 6 months [5]. However, mobile phones do not have the same fate, with phone upgrades occurring approximately every 3 years, perhaps less frequently among lower-resourced communities [6]. Moreover, users can automatically port apps between phone upgrades, allowing long-term tracking for multiple years if there is a reason to want to use the app. Therein lies the final barrier, with the vast majority of apps not being used after just a few months, with the exception of entertainment-based apps [7]. Therefore, gamification of personal data has been lauded as a possible solution [8]. In this work, we present the process of designing a system for and with low socioeconomic status disparity populations through an agile approach, with a particular focus on cardiovascular diseases (CVDs).

CVDs, including coronary heart disease and stroke, remain the leading causes of death, disability, and economic burden globally [9,10]. In 2016, an estimated 17.9 million people died of CVDs, representing 31% of all global deaths [8]. Approximately 85% of CVD-related deaths are attributed to heart attacks or strokes, and Americans experience 1.5 million heart attacks and strokes per year [11]. In these numbers, an alarmingly disproportionate rate of heart-disease incidence is seen among the African American community. Although the national age-adjusted rates of heart attacks and strokes have declined, the rates for African Americans remain 20% higher for heart attacks and 40% higher for strokes, compared with White people [12]. In fact, in the United States, the age-adjusted mortality rate for CVDs is the highest among African Americans compared with all racial and ethnic groups [13]. Modifying behavior and lifestyle choices surrounding physical activity, diet, smoking, sleep quality, and alcohol consumption can significantly reduce the likelihood of developing CVD risk factors such as hypertension, diabetes mellitus, and obesity. Although the improved understanding and control of behaviors associated with CVD risk factors have led to a decline in CVD-related mortality rates in the United States, CVD risk factors manifest earlier in African Americans than in other subgroups, leading to higher mortality rates in the African American community [14].

Advancements in digital technology have changed how people interact with the world and present an opportunity to monitor and modify behaviors that are directly associated with CVD risk factors. Innovations in mobile health (mHealth) technologies and technologies for information and communication technologies have shown promise and evidence for the support of behavior change. For instance, several studies have assessed the impact of mHealth technologies and technology-based (eg, websites, SMS text messaging, telemonitoring, and telemedicine) approaches on chronic disease management (secondary prevention). In recent years, there has been a proliferation of apps and technology-based interventions that promote physical activity [15], smoking cessation [15,16], monitoring caloric intake [17], and providing education on health risk factors, with the primary focus being the prevention of disease advancement [18]. However, there is limited research on leveraging technology-based or mHealth interventions to prevent disease, specifically in younger ethnic minority demographics. As CVD mortality rates are higher among African Americans, and CVD risk factors are evident earlier, engaging young-adult members of the African American community in the development of an mHealth platform that shows promise for primordial prevention (ie, to avoid the development of the initial risk factors) is the next step to improving outcomes. Owing to the disproportionately high prevalence of CVDs in African American communities, the risk factors of CVDs and the ubiquity of mobile phones, young African Americans need to be engaged in the system design of a CVD risk-reduction mobile data collection platform.

### Objectives

This study aims to develop a system design for the MOYO mobile data collection platform using the design requirements for an urban African American population. The function of the MOYO mobile platform is to gather longitudinal and granular environmental, psychosocial, behavioral, and clinical data from a population of young African American adults to reduce CVD risk factors.

## Methods

### Overview

Developed initially for psychiatric populations [19-21], this new platform was developed as a generalized extension to address both psychiatric and physiological health, with an augmented user interface to address these issues, with a particular focus on extending to cardiovascular health.

The study design was an electronic cohort study. The study procedures were approved by the institutional review board of the Morehouse School of Medicine.

### Community-Based Participatory Research and User-Centered Design

Community-based participatory research (CBPR) values community-academic partnership and shared leadership in the planning, implementation, evaluation, and dissemination of initiatives. The 9 key principles of CBPR are recognizing the community as a unit of identity; building on strength and resources with the community; facilitating collaborative, equitable involvement of all partners in all phases of the research; integrating knowledge and action for the mutual benefit of all partners; promoting a colearning and empowering process that attends to social inequalities; involving a cyclical and iterative process; addressing health from both positive and ecological perspectives; disseminating findings and knowledge gained to all partners; and involving a long-term commitment [22]. The research team implemented the principles of CBPR in the recruitment and development of MOYO prototypes that addressed community-identified social, structural, physical, environmental, and policy priorities that impact CVD risk factors.

Similar to CBPR, the user-centered design (UCD) approach explicitly engages end users (ie, community) in the development process. The 2 theories depart in applicability: CBPR is focused on the planning and implementation of community-focused research, and UCD is primarily leveraged to develop consumer-oriented products and applications. By combining CBPR principles and a UCD approach, the research team wanted to ensure that the end user would engage in every phase of development, influence the design, and ultimately increase the usability of MOYO.

### Co-design Sessions

A series of event-based recruitment events (ie, HealthTech Events), similar to coding hack-a-thons, were designed and implemented to extract the design requirements from the target population by (1) leveraging community-based participatory and UCD approaches to engage the end user in identifying environmental factors that contribute to behaviors associated with CVD risk factors, (2) leveraging the Health Belief Model to increase the understanding and ownership of the disparity that exists, (3) use design-thinking to explore solutions to problems associated with the sustained use of mHealth technologies (eg, declining retention rates), and (4) aligning identified requirements with the social cognitive theory of mass communication to ensure that the incorporated design requirements produced a prototype that would ensure behavior change.

The Health Belief Model suggests that a personal belief in the threat of an adverse health outcome and the effectiveness of the recommended intervention will predict the likelihood that the person will adopt the behavior. Conversely, the social cognitive theory of mass communication provides an agentic conceptual framework to analyze the determinants and psychosocial mechanisms through which symbolic communication influences human thought, affect, and action. Although the theories have overlapping constructs, these theories do not serve to assess behavior change equally.

### Participants and Mentors

MOYO was initially conceived as a tool to collect CVD primordial prevention data and to monitor the health behaviors of urban African Americans, aged 18 to 29 years. Participants who self-reported the inclusion criteria were invited to participate in the HealthTech Events and subsequently join the study. The inclusion criteria were (1) self-identification as Black or African American, (2) aged between 18 and 29 years, and (3) own an iPhone operating system or Android-based smartphone. Informed consent was obtained electronically from the study participants.

### Settings

Owing to convenience sampling, some HealthTech Events were hosted at Atlanta-based colleges and summer enrichment programs that target ethnic minorities and underserved communities.

The HealthTech Events took place at academic institutions and community sites where non–African Americans had access to the facility. The research team did not want to exclude community members from learning about the lived experiences of those at the greatest risk of CVD because of ethnicity. Furthermore, non–Black or African Americans were not assigned to a design team nor were they permitted to enroll in the study, unless they self-identified as Black or African American. Subsequently, neither the data nor the design concepts of non–Black or African Americans were included in the analysis. However, non–Black or African Americans were not prevented from attending the training sessions, in which the study participants discussed their design concepts.

Participants were recruited to participate in 1 of the 3 health-related events over the course of 18 months. The research team hosted one event every 4 to 5 months.

### Mentors

Public health graduate students and user experience and user interface designers serve as volunteer mentors for HealthTech Events. Each team was assigned 1 to 2 mentors with diverse expertise (eg, user experience or public health and user interface or public health). To ensure that each member had a foundational understanding of public health and the design process, mentors received a 1-hour training before each event. The mentors guided the end user (ie, HealthTech Event participants) through the 6 phases of the design-thinking process to create and pitch prototypes. Each event included 35 to 50 participants, separated into design teams composed of 5 members each, forming a combined total of 25 teams. Although 25 teams existed, 25 prototypes were not created. Prototypes were not completed as teams were not able to complete the design process because of schedule conflicts. Furthermore, several prototypes were lost because of data loss.

### A Cloud-Enabled Health Insurance Portability and Accountability Act–Compliant mHealth Sensing Infrastructure

Through an agile design process [23,24], the development team created the following superset of components for the Android-based version of the app:

1. High-resolution actigraphy from the movement sensor (with sampling frequency varying based on the Android operating system version and phone model).
2. The deidentified location indicates the distance from the most frequented location at which the least movement occurred (eg, home, work, or school).
3. The social network size was assessed from the calls and SMS text messages. The thinking behind this was that most people who are ill use more traditional communication channels. By hashing each phone number, it is possible to look at the variety and consistency of communication, both incoming and outgoing, to identify changes in social dynamics.
4. The type of establishment in which the user dwells (or probability of this activity) is given by the Android API [25].
5. Place names from the Android place API (deidentified using a white list of major consumer chains).
6. Mode of transport (from Google Fit API).
7. Battery recharging behavior (times at which phone was plugged in).
8. Weather, pollution, and food desert levels are based on the user’s geolocation from a cloud-based bespoke server that consumes the DarkSky API (for weather) and AirNow (for pollution).

We also included several self-administered standardized scales, including:

1. *The Patient Health Questionnaire-9*: The 9-item Patient Health Questionnaire is a 9-question self-report tool used to screen, diagnose, monitor, and measure the severity of depression [26,27].
2. *The Kansas City Cardiomyopathy Questionnaire-12*: The 12-item Kansas City Cardiomyopathy Questionnaire is a 12-item heart failure–specific health status questionnaire. It has 4 domains: physical limitations, symptom frequency, social limitations, and quality of life, as well as a summary score that combines the 4 domain scores [28].
3. *The Patient-Reported Outcomes Measurement Information System Global-10*: The 10-item Patient-Reported Outcomes Measurement Information System is a publicly available global health assessment tool that allows the measurement of symptoms, functioning, and health-related quality of life in a wide variety of chronic diseases and conditions [29].
4. *Quality of life, enjoyment, and satisfaction questionnaire*: The quality of life, enjoyment, and satisfaction questionnaire (Q-LES-Q) is a self-report measure designed to enable investigators to quickly obtain sensitive measurements of the degree of enjoyment and satisfaction experienced by subjects in various areas of daily functioning [30].
5. *Subjective Units of the Distress Scale*: The subjective units of the distress scale is a self-administered scale for the emotional intensity of disturbance or distress experienced by an individual [31].
6. *The Posttraumatic Stress Disorder Checklist* [32].

The posttraumatic stress disorder checklist was included to capture the influence of trauma on the study population, which has been shown to be associated with an increased likelihood of cardiovascular events [33], with a disproportionate effect on African Americans [34]. The research team also included the *MoodZoom* survey from the original Automated Monitoring of Severity of Symptoms app and *Mood Swipe*, a 5-point Likert emoticon-based scale of frowny and smiley faces (
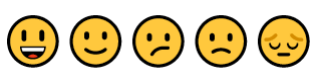
). The latter was implemented as a low friction way of expressing mood in an almost universally accepted manner. Notifications to take the surveys are programmable, with a default to pop up in the morning (between 10 AM and noon depending on the study) and repeated at 2-hour intervals until taken and repeated if ignored for a maximum of 4 times. The start time, frequency, and the maximum number of repeat pop-ups are all programmable. In addition, the user can click on a standard *cog* symbol to alter the types of data collected to adjust the app (as often as they like) to adjust the privacy settings to a level they are comfortable with.

All the above information is synchronized back to the cloud (Amazon Web Services) every 15 minutes and stored in *S3 buckets* in a flat-file data lake organized by study, subject ID, and the week the file was created.

**Figure 1:**
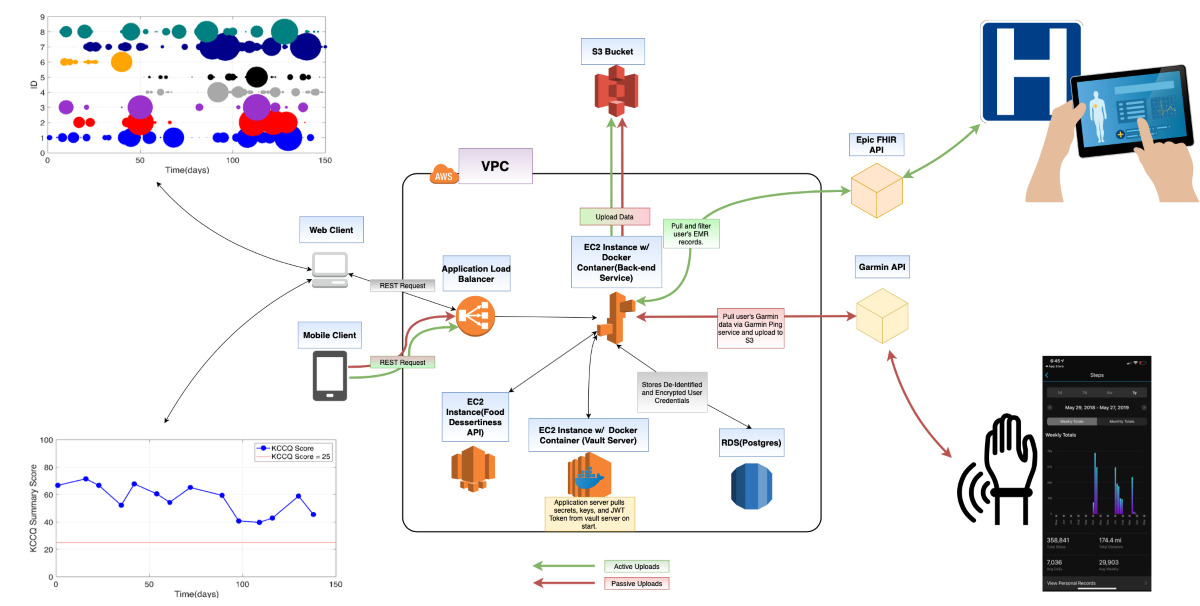
Health Insurance Portability and Accountability Act–compliant cloud-based infrastructure for collecting user input, phone sensor data, wearable technology, and electronic health record data. Upper left: a visualization of social networking behavior. Lower left: daily responses to a standardized questionnaire. Center: the Amazon Web Services cloud infrastructure. Upper right: integration with the electronic health record. Lower right: integration with wearables. API: application programming interface; AWS: Amazon Web Services; EMR: electronic medical record; FHIR: Fast Healthcare Interoperability Resources; JWT: JSON Web Tokens; KCCQ: Kansas City Cardiomyopathy Questionnaire-12; RDS: Relational Database Service; VPC: Virtual Private Cloud.

Electronic health record data are extracted from the electronic health record system using Substitutable Medical Applications, Reusable Technologies on Fast Healthcare Interoperability Resources [35]. The data are then deidentified on our back-end server by dynamically parsing and removing any trace of the subject’s identity. Once deidentified, the data are then associated with the user’s unique study ID before being uploaded to the S3 buckets. Figure 1 illustrates this framework and the design of the cloud infrastructure.

Each day, a *cron job* on Amazon Web Services identifies all the new data and sends out a comma separated value log file via email to the study coordinator to let them know if any individuals have stopped sending data or types of data are not being received. This could easily be adapted to an interface with alerts or a secondary app with pop-up reminders, but email was the preferred medium for the coordinators.

### Pilot Testing

To stress test the infrastructure, a digital US $20 Amazon card was offered to encourage the use of the app. No other incentives or approaches to engaging the community were offered during pilot testing. To assess the utility of the app during pilot testing, the research team measured the number of downloads of the app from Google Play Store, the number of users regularly uploading data (ie, at least 7 times a week), and types of data most frequently uploaded to provide a picture of the most engaging aspects of the app.

### Analysis

From the HealthTech Events, the research team collected 13 prototypes, consisting of 297 screens (ie, each frame of a design is counted as one screen). All prototype screens were uploaded by the mentors to a password-protected shared drive. The prototype screens were analyzed using NVivo (version 12; QSR International), a qualitative analysis software. Using the grounded theory approach, an open-coding method was applied to a subset of data, approximately 20% (5/25) or 5 complete prototypes (5 teams’ complete app design) to identify themes. To ensure intercoder reliability, 2 research team members analyzed the same subset of data, ensuring that the emerged themes were consistent. These themes were used to develop a codebook that guided the analysis of the remaining data. Each prototype screen was tagged with the corresponding theme.

## Results

### Overview

**Figure 2:**
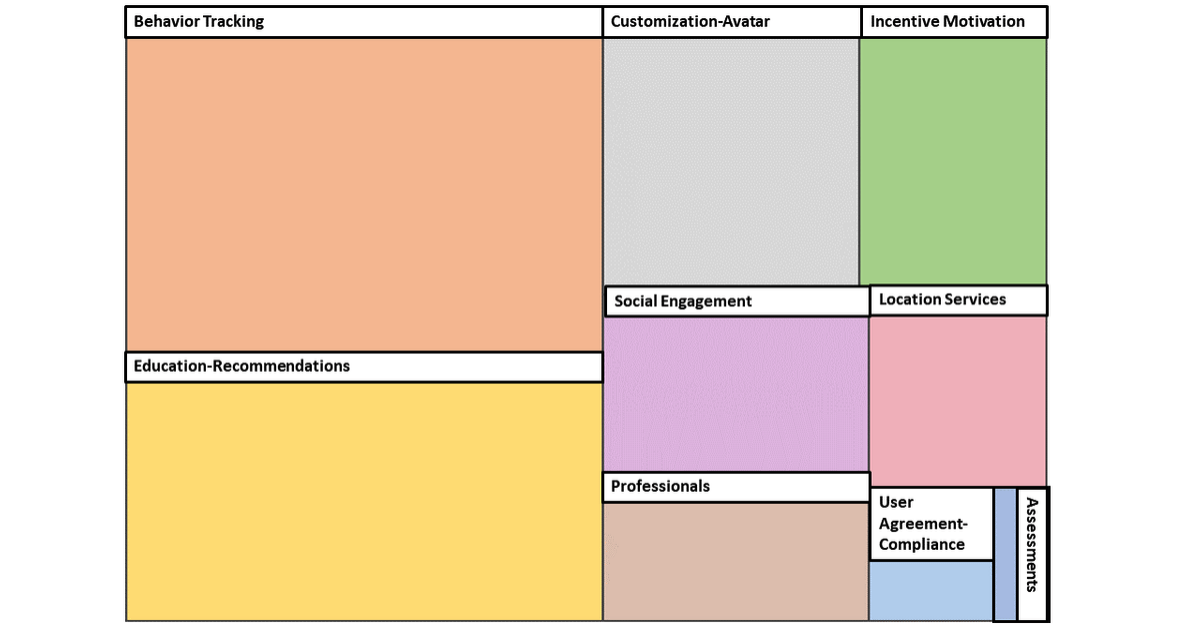
Theme comparison diagram: box size corresponds with the frequency of prototype themes in the analysis.

A total of 9 themes were identified from 297 prototype screens as images. Table 1 outlines the emerging themes, corresponding subthemes, descriptions, and frequencies. Figure 2 indicates the frequency; the more significant the box, the more frequent the theme emerged. The findings reveal the factors that the target audience would find most engaging in an app designed to deliver primordial prevention strategies aimed at reducing CVD risk. The elements that emerged can be organized into 4 overarching categories: individual, interpersonal, expert-informed, and technological. These findings are described in the following sections.

**Table 1:** User-centered design session thematic analysis and frequency of themes.

Theme			Frequency (N=297), n		Description
**Behavior tracking**			74		
	Exercise			Record physical activity	
	Diet			Maintain a food journal	
	Mental health			Record mood and stress (self-reported)	
**Education and recommendations**			57		
	Push messaging			Receiving recommendations to improve deficient areas (ie, tips to improve sleep quality)	
	Healthy tips			Provision of healthy recipes and/or healthy meal options when eating out	
	Health considerations			Provide baseline health information (ie, importance of cardiovascular health, BMI, and exercise frequency recommendations)	
**Customization**			33		
	Textures			Color scheme, background, and font	
	Integration			Sync with other apps (ie, Spotify and iTunes)	
	Avatar			Visual depiction of oneself	
**Incentive motivation**			24		
	Competition			Peer-to-peer or group challenges	
	Leaderboards			Incorporation of a scoreboard and/or ranking system	
	Rewards			Discounts and point accumulation for reaching goals	
**Social engagement**			22		
	Social network integration			Ability to sync social networking sites	
**Health professionals**			17		
	Telehealth and telepsychiatry			Ability to interact (ie, video and SMS text messaging) with a health professional	
**Location services**			16		
	Diet			Leverage location settings provide health food options	
	Fitness			Provide nearby physical activity opportunities (ie, exercise classes, park, and track)	
**User agreements**			7		
	User security			Consent to data use	
**Assessments**			3		
	Mental health			In-depth screening of mental health; survey	

### Individual Factors

#### Customization

Several end users designed their prototypes with features that would allow for a degree of personalization of the app, including selecting themes (color, font, and commonly used features), syncing other apps (iTunes and Spotify), and creating a visual representation (avatar) within the app. The avatar design varied in functionality and utility, ranging from a mere visual depiction to displaying apparent weight and energy changes based on the data collected (ie, food consumed and biometric entries).

#### Behavior Tracking

Most of the prototypes included tracking behaviors associated with CVD risk factors (diet, exercise, sleep, and mental health). In addition to tracking these behaviors, several designs also included goal-setting features. Thus, end users would be able to determine benchmarks, reflect on accomplishments, and identify challenge areas. In many prototypes, behavior-tracking features integrated other apps or leveraged technology to automate data collection and reduce end user burden. For example, to create a food journal entry, the end user can take a picture of their meal instead of entering each item they ate.

### Interpersonal Factors

#### Incentive Motivation

End users incorporated an array of features that incentivized consistent positive behaviors, including promoting *friendly competition*, leaderboards, and a reward system for accomplishing goals. Several prototypes encouraged the end user to engage their peers by challenging them to attain the set goal. In some designs, the end user could join challenges with other users that they did not know and compete to reach the same goal. Furthermore, end users could also be rewarded for accomplishing their individual goals, such as receiving a coupon for logging health meals for a week.

#### Social Engagement

Many prototypes were built to interface with social networking sites (Snapchat, Instagram, and Twitter). The end user could share their progress on social media and encourage peers to join challenges.

### Expert-Informed Factors

#### Education and Recommendations

Several prototypes include features that would allow user-specific education and recommendations. The information and recommendations provided are extensions of the self-reported behavior tracking. On the basis of the data entered in the app, end users could receive information to improve or increase activity in certain areas. For example, if the user data shows that the user has poor sleep quality, then using push messaging or prompts after entering the app, the user is provided with information about the relationship between sleep and cardiovascular health and tips to improve sleep quality.

#### Health Professionals and Assessments

A critical feature included in some prototypes was real-time interaction with health professionals. Leveraging telehealth technology, end users can connect with professionals regarding health-related concerns. The communication style with professionals included text messages, video calling, and store-and-forward video messaging. Some design elements were specific to mental health. A few prototypes included a self-reported assessment that screened mental well-being based on mood or responses to external factors (job, stress, and family). On the basis of the end user responses to the evaluation, the user may be advised to talk with a professional. In addition, end users had the option to engage with both mental and physical health professionals at any point.

### Technological Factors

#### Location Services

Prototypes leveraged the end user location to provide user-specific diet and fitness recommendations. End users were able to identify the local healthy food options. In addition, location features could encourage users to engage in nearby physical activity opportunities (ie, fitness classes and upcoming races).

#### User Agreements

Prototypes provide end users with easy-to-understand data security agreements. The end users desired the ability to make an informed decision regarding the data shared with the research team and how the research team will use the data.

### Final Design

**Figure 3:**
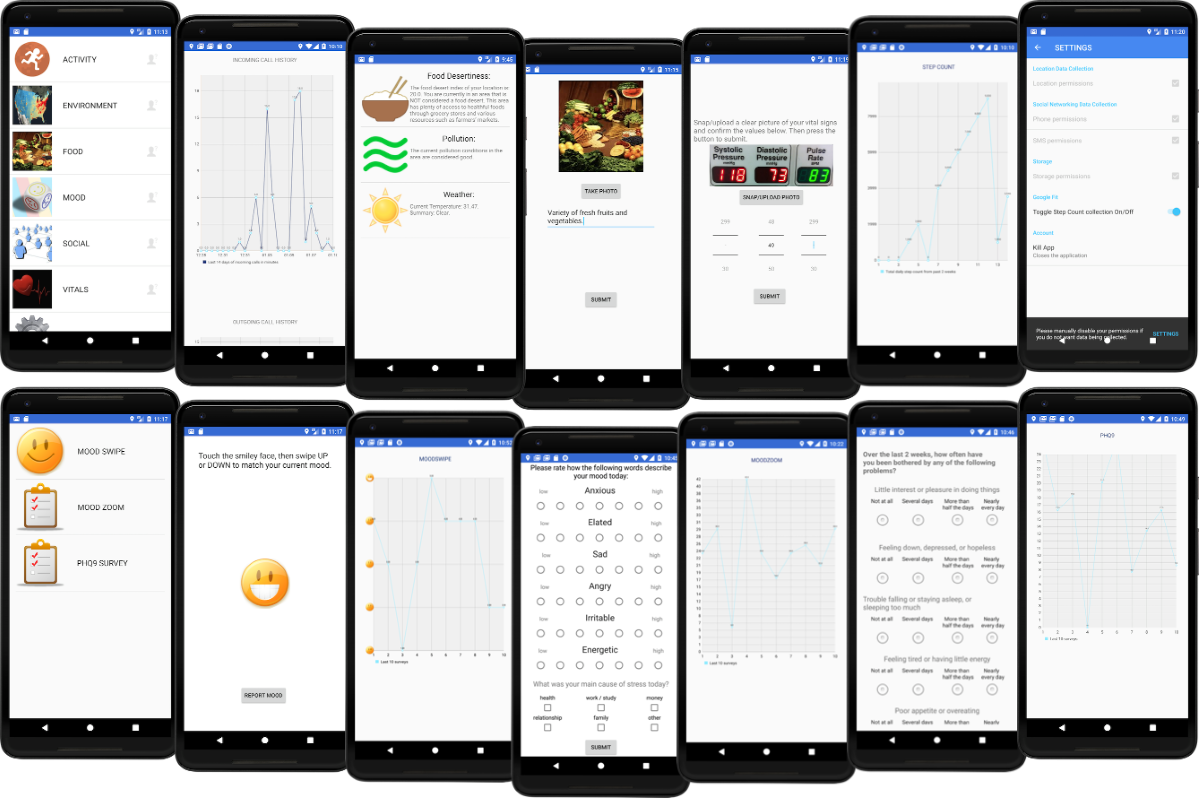
Mobile data collection platform prototype design. The 6 major categories of data collected were physical activity, environment, food, mood, social behavior, and physiology (vitals), driven by a main menu (top left screenshot) and represented by 6 different interfaces (second from left to end in the top row).

Figure 3 illustrates the design of the resultant interface. The app covers 6 major categories.

1. Physical activity: The user can track how much they have moved during the time they have been carrying the phone. Historical graphs provide insight into the regularity of exercise (conscious or otherwise). In the iPhone operating system, this is tracked through the nonscientific notion of *steps* for the lack of another solution. This is only for relative movement information.
2. Environment: Using external APIs, the app collects frequent data (every hour or 100 m moved) on pollution, weather, and food desert indices.
3. Food: Through photographs and diaries, a user can document food consumption
4. Mood: Through standardized, digital self-administered forms, psychological health can be assessed and tracked.
5. Social behavior: The complexity and dynamics of an individual’s social network give a perspective on life changes that may affect how their mood changes and help identify positive and negative influences in life.
6. Physiology or vitals: Through photographs of medical devices, vital signs such as blood pressure can be logged at the time of collection. Future work will focus on autotranscribing readings, as demonstrated in [36].

### Pilot Test

Following the design and deployment of the app through the Google Play and Apple app stores, the app was downloaded 181 times during the 24 months, with the bulk being around the HealthTech Events. A total of 116 users regularly uploaded data, with an average of 277 uploads per week. The volume of data uploaded depended on the behavior of the user and whether there was a loss of network connectivity for any extended period (as the missed uploads are synchronized when connectivity is restored). If the user does not move, enter data, or interact with the phone, a lower volume of data is collected. The average payload upload size was 0.024 MB per person per day.

**Figure 4:**
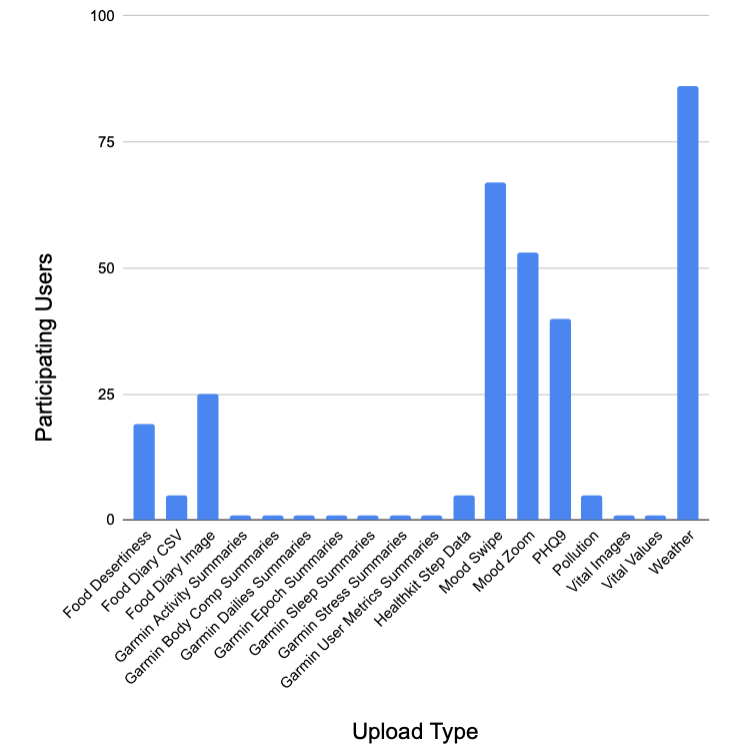
Histogram of the number of users uploading each data type. Most users did not engage with the wearables (which were optional), but both passively collected data and self-reported mental health surveys were often uploaded. PHQ-9: Patient Health Questionnaire-9

Figure 4 illustrates the relative proportion of upload types throughout the pilot, with passive geolocation-triggered data (weather and food dessert index) being among the most frequently uploaded. However, active self-reported mood (through 3 separate surveys: mood swipe, mood zoom, and the Patient Health Questionnaire-9) were as frequent, indicating that the app was engaging in tracking mental health.

## Discussion

### Principal Findings

An extensive app-cloud infrastructure was developed, which provided detailed information on the physical activity, behaviors, and psychosocial and physiological state of urban African American young adults. The research team followed a community-based participatory design approach that provided a simple method for entering or sourcing information that the community considered essential to engaging with the app. As the system design is flexible, the MOYO mobile platform has broad applicability. Furthermore, because the system is constructed around a cloud platform, the analysis can be automated and scaled.

Integration with wearables has been demonstrated through a proof of concept, but we still warn users against the utility of crowdsourcing quasi-medical data, without standardization. For example, in all our research on pregnant women, blood pressure data capture is standardized to the use of Omron M7, which is validated in preeclampsia. Small changes in the capture protocol, cuff form factor, or device firmware could significantly change the readings. For example, switching to a different version of the same device halfway through a clinical trial (perhaps because the original device went off-market) could lead to a slight offset across the population after intervention, making the trial seem successful when it was not, or vice versa. Of course, such issues are true for any device, but this problem is particularly exacerbated when using devices that can be updated over the air, such as commercial fitness devices or smartwatches. If the study coordinator or user does not set the device to prevent updates, changes in the device behavior will occur in a somewhat arbitrary manner (depending on when the update is issued and when and if the user decides to accept the update). Even then, devices issued later in a study could carry a later version of the firmware, unknown to the study team.

In an earlier study, where we used the Jawbone UP3 fitness and sleep tracker, we worked with the company to whitelist the study devices to ensure that the company did not update the firmware for the device at any point [21]. We also set the phones to prevent updating Android operating system versions as this can change the resolution of the location and sampling frequency of the accelerometer. We note that the one algorithm over which we have no control is the type of building returned by Google’s Map API. However, as retail establishments change over time and maps can be noisy, such information should be treated with caution, and a crowdsourcing approach to relabeling data may be applicable. To some extent, we cannot control any changes in the weather API either, but this is less of a concern because we do not expect the measurement of temperature, humidity, and pollution to change significantly over time. Moreover, the weather is a relatively coarse concept applied over a wide area, heavily averaged, and interested users can pull historical data from multiple weather APIs to improve accuracy. To mitigate these issues, we suggest relative changes, rather than absolute changes, as features or flags for changes in health and behavior. In terms of engagement, both passive (eg, weather and food desert) data were frequently uploaded and active reports on mental health, whereas wearable-based activity data showed little uptake. This is perhaps not surprising in a young-adult cohort, where perceived barriers and facilitators to mental health help-seeking in young adults occupy more of their time than concerns over physical well-being [37].

### Conclusions

Although we have demonstrated some successes with the use of the app in CVD populations, the potential to shift the needle with young adults who are yet to perceive a health problem is still problematic. As noted above, gamification and use of behavioral economics are perhaps keys to the retention of users and the ability of the information to affect changes in user lifestyles without chronic or acute issues at the start of their journey turning into illness or wellness. However, without strong community participation and ownership of the system, we cannot expect deep engagement. Thus, the findings from this study support a community-based participatory design approach to solve the problems of health inequities. More specifically, African Americans should be meaningfully involved in the design of technologies that are developed to improve CVD outcomes within African American communities.

## Abbreviations

API: application programming interface
CBPR: community-based participatory research
CVD: cardiovascular disease
mHealth: mobile health
UCD: user-centered design
